# Differential gene expression and pathway analysis in growth hormone-secreting pituitary tumors according to granulation pattern

**DOI:** 10.3389/fonc.2024.1423606

**Published:** 2024-07-30

**Authors:** Kyungwon Kim, Yeongmin Kim, Se Hoon Kim, Ju Hyung Moon, Eui Hyun Kim, Eun Jig Lee, Chang-Myung Oh, Cheol Ryong Ku

**Affiliations:** ^1^ Endocrinology, Institute of Endocrine Research, Department of Internal Medicine, Yonsei University College of Medicine, Seoul, Republic of Korea; ^2^ Department of Biomedical Science and Engineering, Gwangju Institute of Science and Technology, Gwangju, Republic of Korea; ^3^ Department of Pathology, Severance Hospital, Yonsei University College of Medicine, Seoul, Republic of Korea; ^4^ Department of Neurosurgery, Severance Hospital, Yonsei University College of Medicine, Seoul, Republic of Korea

**Keywords:** growth hormone-secreting pituitary tumor, acromegaly, sparsely granulated somatotroph adenoma, densely granulated somatotroph adenoma, granulation patterns

## Abstract

This study investigated differential gene expression between granulation patterns in growth hormone (GH)-secreting pituitary tumors, aiming to elucidate novel transcriptomes that explain clinical variances in patients with acromegaly. Transcriptome analysis was conducted on 6 normal pituitary tissues and 15 GH-secreting pituitary tumors, including 9 densely granulated somatotroph tumors (DGSTs) and 6 sparsely granulated somatotroph tumors (SGSTs). We identified 3111 differentially expressed genes (DEGs) in tumors compared to normal pituitaries, with 1117 DEGs unique to a specific granulation within tumors. SGST showed enrichment of neuronal development and acute inflammatory response pathways, along with a significant enhancement of JAK–STAT, phosphatidylinositol 3-kinase, and MAPK signaling. The results suggest that granulation-specific gene expression may underpin diverse clinical presentations in acromegaly, highlighting the potential for further investigation into these transcriptomic variations and their roles in disease pathology, particularly the involvement of genes linked to neuronal development, inflammatory response, and JAK–STAT signaling in SGST.

## 1 Introduction

Growth hormone (GH)-secreting pituitary tumors, a rare yet incurable disease, significantly contribute to acromegaly, increasing mortality by over 15-fold due to heightened morbidity from cardiovascular, metabolic, and malignant diseases (1, 2). The treatment objectives for acromegaly aim to normalize biochemical markers, eradicate or manage tumor mass while preserving normal pituitary function, alleviate symptoms, and ultimately align life expectancy with that of the general population (3). Surgical resection is recommended as the primary therapy for GH-secreting pituitary tumors, with cure rates ranging from 40 to 70%, depending on tumor size (4). Notably, if patients do not respond to surgery, medical treatment is pursued (5–7). However, postoperative pharmacotherapy and radiotherapy yield only a 60% cure rate. Additionally, patients with drug-resistant acromegaly exhibit a variable medical treatment response rate of 40–65% (7).

The 2022 World Health Organization (WHO) guidelines classify GH-secreting pituitary adenomas into densely granulated somatotroph tumors (DGSTs) and sparsely granulated somatotroph adenomas (SGSTs) based on GH immunoreactivity and cytokeratin patterns (8). SGST is predominantly characterized by a fibrous body pattern (> 70%), whereas DGST is identified by a perinuclear cytokeratin pattern, with or without focal fibrous bodies (9).

Clinically, the densely and sparsely granulated types exhibit contrasting phenotypes regarding hormone secretion and drug response. DGST significantly elevates growth hormone and insulin-like growth factor 1 (IGF-1) levels compared to the sparsely granulated type (10). SGSTs are larger in size and exhibit more aggressive behavior (11, 12). Notably, 50% of patients have densely granular tumors, whereas 30% present with sparsely granular tumors, which are more prevalent in younger patients (<50 years) and show less responsiveness to somatostatin receptor ligands (SRLs) (12–14). Densely granulated adenomas, which display a higher number of cells reactive to E-cadherin and β-catenin, indicate a more differentiated phenotype (9). The mechanisms driving these behavioral differences in tumors remain unclear. Up to 50% of densely granulated adenomas carry a *gsp* mutation that activates cAMP, providing an intracellular target for somatostatin therapy (11). Conversely, some sparsely granulated tumors exhibit reduced somatostatin receptor expression and mutations in the GH receptor’s extracellular domain, contributing to SRL resistance (15).

To date, various clinical factors linked to the drug responsiveness of GH-secreting pituitary tumors have been identified, which are often associated with their granulation patterns (densely vs. sparsely) (12). The molecular mechanisms differentiating the morphological subtypes of GH-secreting tumors are only partly understood and do not fully account for the variable behaviors of these subtypes. Uncovering distinct mechanisms and pathways between the two subtypes, along with new molecular biomarkers, may improve disease prognosis and lead to more effective therapeutic targets.

High-throughput RNA sequencing has proven highly beneficial for precision medicine, linking genotypes to phenotypes and elucidating the pathogenesis of pituitary tumors (16, 17). This study aimed to identify genes that are differentially expressed between granulation patterns and highlight key transcriptomes associated with clinical differences in patients with GH-secreting pituitary tumors.

## 2 Materials and methods

### 2.1 Participants

This study was approved by the Ethics Committee of Severance Hospital, and informed consent was obtained from all participants (IRB No. 4–2011-0740). We performed high-throughput RNA sequencing on twenty GH-secreting pituitary tumors from consenting participants and 6 normal pituitary glands provided by the National Forensic Service in Gangwon-do, South Korea. Criteria for selecting individuals for normal pituitary gland samples included no preexisting medical conditions and death due to acute heart disease. All normal tissues were collected within 24 hours of death after excluding pituitary incidental tumors by H&E staining. The median age of the participants for normal pituitary tissues was 54 years (ranging from 50 to 75 years), with 50.00% of them being female. Immunohistochemical staining of the operated pituitary tumors was performed for GH, prolactin, luteinizing hormone, follicle-stimulating hormone, thyroid-stimulating hormone, and adrenocorticotropic hormone. Pituitary tumors expressing hormones other than GH were excluded from this study. Finally, our study included 15 patients after excluding two who did not fit into either the DGST or SGST categories. Ultimately, 15 somatotroph adenomas, comprising 9 DGSTs and 6 SGSTs, along with 6 normal pituitaries, were analyzed for transcriptomic differences.

All patients diagnosed with acromegaly, confirmed using a 75 g oral glucose tolerance test (OGTT) and sellar magnetic resonance imaging (MRI), were enrolled in this prospective cohort study. Patients had undergone TSA from 2013 to 2020 and consented to sample collection and analysis. Clinical data, including age and sex, were extracted from electronic medical records. Pathological results, including those from electron microscopic evaluations, were also collected.

### 2.2 Endocrinological and imaging studies

Serum GH levels were monitored at 0, 60, 90, and 120 min following the ingestion of 75 g of oral glucose during the 75 g OGTT. Serum IGF-1 levels were also measured each time a 75 g OGTT was conducted. The OGTTs were performed both at the time of diagnosis and six months post-TSA. GH and IGF-1 levels were assessed using a chemiluminescence immunoassay (CLIA, LIASON by DiaSorin, Saluggia, Italy). IGF-1 concentrations were expressed as a fraction of the upper limit of normal (ULN) of the reference ranges. Biochemical remission was defined as a GH level of <1.0 ng/mL during the follow-up 75 g OGTT at six months. Tumor size and cavernous sinus invasion were evaluated based on a sellar MRI. Various 3.0-Tesla MRI units (Discovery MR750/750, GE Healthcare or Achieva/Ingenia/Ingenia CX, Philips Medical Systems) were utilized for this purpose. We applied the Knosp classification to categorize parasellar growth into five grades.

### 2.3 Tissue collection and histopathological analysis

Pituitary tumor tissues resected via TSA were fixed in 10% buffered formalin overnight. Sections from these paraffin-embedded blocks were stained using hematoxylin and eosin, Gomori’s reticulin, and periodic acid-Schiff-orange G histochemical stains. Immunohistochemical staining was performed for anterior pituitary hormones, including follicle-stimulating hormone, luteinizing hormone, thyroid-stimulating hormone, adrenocorticotropic hormone, prolactin, and GH, utilizing the peroxidase–anti-peroxidase complex technique with light hematoxylin counterstaining. Transcription factors PIT-1, steroidogenic factor 1, and T-box family member TBX19 were also evaluated. According to the 2022 WHO classification (8), all tissues exhibited positive staining for GH and PIT-1. Ki-67 index in post-surgical specimens was measured via IHC using the MIB-1 antibody (Dako Denmark A/S, Glostrup, Denmark). A specialized neuropathologist counted the positively stained nuclei in hotspot areas and expressed the Ki-67 levels as a percentage of total tumor cells. The Ki-67 index was analyzed using 3% as the cutoff value (18, 19).

### 2.4 Electron microscopic evaluation

Electron microscopy (EM) studies of GH-secreting pituitary tumors have been conducted at the Department of Pathology in Severance Hospital since 2018. The specimens were fixed for 2 h in a solution of 2% glutaraldehyde and 2% paraformaldehyde in 0.1 M phosphate buffer (pH 7.4), followed by washing in the same buffer. They were then post-fixed with 1% osmium tetroxide in 0.1 M phosphate buffer for 2 h and dehydrated in an ascending series of ethanol concentrations (50%, 60%, 70%, 80%, 90%, 95%, and 100%) for 10 min each, followed by a 10-min infiltration with propylene oxide. The specimens were embedded using a Poly/Bed 812 Kit (Polysciences) and polymerized in an EM oven (TD-700, DOSAKA, Japan) at 65°C for 12 h. The block, equipped with a diamond knife in an ultramicrotome, was sectioned into 200 nm semi-thin slices and stained with toluidine blue for optical microscope examination. The regions of interest were then sectioned into 80 nm thin slices using an ultramicrotome, placed on copper grids, double-stained with 3% uranyl acetate for 30 min and 3% lead citrate for 7 min, and imaged with a transmission electron microscope (JEM-1011, JEOL, Tokyo, Japan) at an acceleration voltage of 80 kV, equipped with a Megaview III CCD camera (Soft Imaging System, Germany).

Somatotroph adenomas were semiquantitatively classified by a special neuropathologist based on the following criteria: (i) DGST, when perinuclear pattern cells exceeded 70% and dot-like pattern cells were <10%, regardless of the percentage of transitional pattern cells; (ii) SGST, when dot pattern cells exceeded 70%, irrespective of the percentages of perinuclear and transitional pattern cells; and (iii) the transitional type, when the sample did not meet the criteria for the aforementioned categories (9).

### 2.5 Data pre-processing

The quality of RNA seq *reads (*fastq files) was confirmed using the FastQC(version 0.11.9) (Babraham Bioinformatics, Cambridge, UK, https://www.bioinformatics.babraham.ac.uk/projects/fastqc/). The adaptor sequences were removed from the raw data using the Trimmomatic (version 0.36) (20). The sequences were aligned to the Human reference genome NCBI GRCh38.109 using STAR (version 2.7.10) (21). The duplicated sequences were removed using Picard’s MarkDuplicates function (version *3.0.0) (*“Picard Toolkit.” 2019. Broad Institute, GitHub Repository. https://broadinstitute.github.io/picard/; Broad Institute). The Sequence Alignment/Map files were converted to binary form using Samtools (version 1.6) (22). The transcript abundance was calculated using the HTseq (version 2.0.2) (23).

### 2.6 High-throughput total RNA sequencing

To construct cDNA libraries, we used 0.5 μg of RNA with the Illumina TruSeq Stranded Total RNA Library Prep Gold Kit (Illumina, Inc., San Diego, CA, USA, #20020599). The library concentrations were measured by a quantitative polymerase chain reaction using the TapeStation D1000 ScreenTape (Agilent Technologies, #5067–5582). These indexed libraries were then processed using the Illumina NovaSeq system (Illumina) for paired-end sequencing (2 × 101 bp), conducted by Macrogen in Seoul, Korea. Subsequently, the output data were demultiplexed using bcl2fastq version 2.2, which generated FastQC files.

### 2.7 Principal component analysis

Each gene in the sparse, dense, and normal tissues included various dimensions. To visualize gene clusters, dimensionality reduction was conducted using principal component analysis (PCA). The results of PCA were visualized using ggplot2 in R. The PCA plot indicates that PC1 and PC2 are x- and y-axes, respectively, and colors indicate each sample condition.

### 2.8 RNA sequencing analysis

To normalize the count data calculated by HTseq, DEseq2(version 1.40.2) was conducted (24). lfcShrink function was also conducted to improve biostatistics dispersion. DEGs, with p-adjusted value < 0.05 and |log2FC|>1, were extracted from normalized count data and used for further studies. The DEGs in Acromegaly vs Normal and Sparsely vs Densely groups were visualized using quadrants and volcano plots, respectively. The genes in Acromegaly vs Normal and Sparsely vs Densely groups were visualized using EnhancedVolcano (version 1.18.0); the gray, red, and blue dots indicated non-DEGs, Up-DEGs, and Down-DEGs, respectively.

### 2.9 Gene ontology and gene set enrichment analyses

To identify comprehensive alteration in Acromegaly and Normal pituitary and Sparsely and Densely adenocarcinoma, Gene Ontology (GO) and Kyoto Encyclopedia of Genes and Genomes (KEGG) pathway enrichment analyses were conducted using ClusterProfiler (version 4.8.3) (25). The genes used in GO enrichment analysis were DEGs with p-adjusted values < 0.05 and |log2FC| > 1. To analyze the metabolic alteration in acromegaly, only the biological process term was used. The results of GO and KEGG enrichment analyses were visualized as a bar plot; the colors indicate the p-adj value of the GO terms. The red and blue bars indicate GO terms associated with up- and downregulated DEGs, respectively.

To analyze the metabolic processes associated with Sparsely vs. Densely, we used the Gene Set Enrichment Analysis (GSEA) provided by ClusterProfiler, with a p-adj value cutoff < 0.05. Only BP terms were utilized in our study. The GSEA results were visualized using enrichplot (version 1.20.1) (26).

### 2.10 Protein-protein interaction

To find out the hub genes, act as important intermediate pathways, PPI analysis was conducted for DEGs with p-adjusted values < 0.05 and |log2FC| > 1 between SGST and DGST. We excluded DEGs with fewer than 5 PPI because theses DEGs were not suitable for hub genes. The protein-protein interaction (PPI) database was obtained from the BioGRID (https://thebiogrid.org/) (27), The Human Reference Interactome (http://www.interactome-atlas.org/), IntAct (https://www.ebi.ac.uk/intact/home) (28), HitPredict (http://www.hitpredict.org/), Integrated Interactions Database (https://iid.ophid.utoronto.ca/), The Molecular INTeraction Database (https://mint.bio.uniroma2.it/) (29), Search Tool for the Retrieval of Interacting Genes/Proteins (https://string-db.org), Database of Interacting Proteins (https://dip.doe-mbi.ucla.edu/dip/Main.cgi) (30), Human Protein Reference Database (https://www.hsls.pitt.edu/obrc/index.php?page=URL1055173331), and the heterologous Interaction Database (31). The database’s physical interactions or experimentally verified interaction data are only used to predict PPIs. The results of the PPI network were visualized using Cytoscape software version 3.10.1. The string indicates a PPI. The nodes indicate proteins. The red and blue colors indicate up- and downregulated DEGs, respectively. To find out the top ten Hub genes, “cytoHubba”(version 0.1) was used.

### 2.11 Statistical analyses

Values are expressed as means (standard deviation) or medians (range). We compared values between two groups using Student’s t-test for parametric values and the Mann–Whitney U test for non-normally distributed numeric variables. The Kruskal–Wallis H test was used to compare three groups for fragments per kilobase of transcript per million mapped reads from RNA sequencing data. For categorical data, the Chi-square test or Fisher’s exact test was employed. Statistical analyses were conducted using SPSS software (SPSS, version 22; Chicago, IL, USA). P-values < 0.05 were considered statistically significant.

## 3 Results

### 3.1 Clinical characteristics of patients

**Table 1** presents the baseline characteristics of the patients who underwent RNA sequencing. The study included 15 patients with an average age of 50.00 years at diagnosis (range: 28.00–64.00 years), of which 60.00% were female. The median initial levels of GH and IGF-1 were 42.50 ng/mL (range: 3.50–80.00 ng/mL) and 2.97 xULN (range: 0.76–4.00 xULN), respectively. Surgical remission post-transsphenoidal adenectomy (TSA) was achieved in 80.00% of the patients.

**Table 1:** Clinical characteristics of patients with GH-secreting pituitary tumors by granulation pattern.

	Total N = 15	DGST N = 9	SGST N = 6	P value
Age at diagnosis (years)	50.00 (28.00–64.00)	56.00 (28.00–64.00)	43.00 (37.00–56.00)	0.087
Women, n (%)	9/15 (60.00%)	5/9 (55.56%)	4/6 (66.67%)	1.000
Preoperative random GH (ng/mL)	42.50 (3.50–80.00)	47.50 (3.50–76.90)	31.35 (6.20–80.00)	0.906
Preoperative nadir GH (ng/mL)	18.00 (2.20–80.00)	22.80 (2.20–58.90)	17.15 (4.70–80.00)	0.637
Preoperative IGF-1 (xULN))	2.97 (0.76–4.00)	2.97 (0.76–4.00)	2.99 (2.35–3.85)	0.346
Tumor size (mm)	19.00 (12.00–45.00)	16.00 (12.00–27.00)	20.00 (14.00–45.00)	0.236
Cavernous sinus invasion, n(%)	7/15 (46.67%)	3/9 (33.33%)	4/6 (66.67%)	0.315
Surgical remission, n (%)	12/15 (80.00%)	7/9 (77.78%)	5/6 (83.33%)	1.000
Knosp grade				
0	1/15 (6.67%)	1/9 (11.11%)	0/6 (00.00%)	0.144
1	6/15 (40.00%)	5/9 (55.55%)	1/6 (16.67%)	
2	1/15 (6.67%)	0/9 (0.00%)	1/6 (16.67%)	
3a/b	5/15 (33.33%)	3/9 (33.33%)	2/6 (33.33%)	
4	2/15 (13.33%)	0/9 (00.00%)	2/6 (33.33%)	
Ki67 index, n (%)				
<3.0	14/15 (93.33%)	9/9 (100.00%)	5/6(83.33%)	0.400
≥3.0	1/15 (6.67%)	0/9(0.00%)	1/6(16.67%)	

GH, growth hormone; DGST, densely granulated somatotroph tumor; SGST, sparsely granulated somatotroph tumor; IGF-1, insulin-like growth factor 1. Data are presented as median ± SD.

Immunohistochemical analysis confirmed the presence of GH and PIT-1 in all adenomas. Based on electron microscopic examination, patients with acromegaly were categorized into DGST or SGST groups. Clinical and metabolic measurements were recorded for both subtypes: 9 DGSTs and 6 SGSTs. According to **Table 1**, the median tumor size was 19.00 mm (DGST: 16.00 mm; SGST: 20.00 mm). No statistically significant differences were observed between the two groups in terms of hormone levels, surgical remission rates, tumor sizes, cavernous sinus invasion and Ki67 index.

### 3.2 Transcriptome profile of GH-secreting pituitary tumors

**Figure 1:**
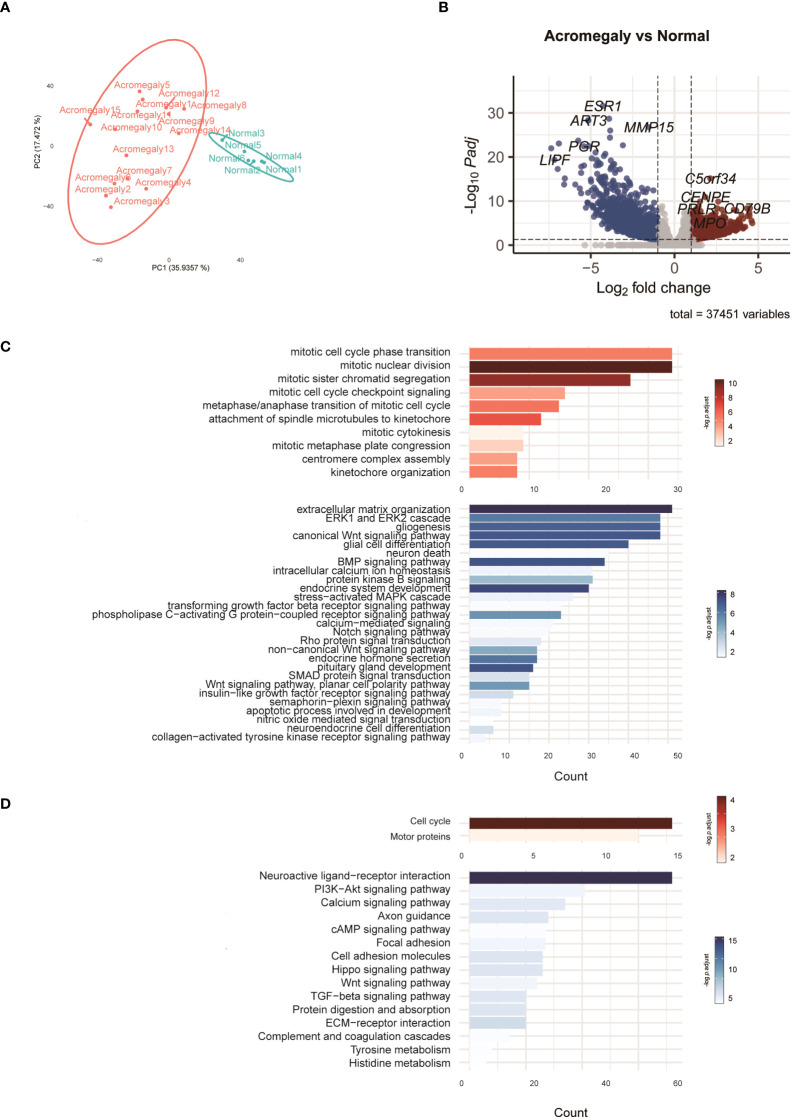
Characteristics of the transcriptomes in GH-secreting pituitary tumors compared to normal pituitaries. **(A)** PCA of the RNA-seq normalized counts of acromegaly and normal. **(B)** Volcano plot visualizes acromegaly DEGs. The red, blue, and grey dots indicate upregulated, downregulated, and non-DEGs, respectively. **(C)** The results of the GO analysis are indicated by a bar plot. The red and blue bars indicate upregulated and downregulated GO terms in GH-secreting pituitary tumors, respectively. **(D)** The results of KEGG are indicated by a bar plot. The red and blue bars indicate up and downregulated pathways, respectively.

We analyzed the gene expression of GH-secreting pituitary tumors and normal pituitary tissues. **Table 2** lists the top genes that were differentially expressed in GH-secreting pituitary tumors relative to normal pituitaries. **Figure 1** illustrates genes that differentially exhibited changes in expression in tumor tissues compared to those in normal tissues. The transcriptomic data revealed numerous DEGs, with 1794 downregulated and 1317 upregulated genes in GH-secreting adenomas relative to normal pituitary tissues, using a threshold of |log2 fold change| > 1 and an adj P-value < 0.05 (**Figure 1B**).

**Table 2:** Representative differentially expressed genes (DEGs) in GH-secreting pituitary tumors compared with those in normal pituitaries.

Condition	Gene	Log2FC	Adjusted P value	Description
Up	*CHRNA6*	4.594485736	2.60E-06	cholinergic receptor nicotinic alpha 6 subunit
	*PBK*	4.496781402	4.03E-09	PDZ binding kinase
	*ELFN2*	4.473790232	2.06E-07	extracellular leucine-rich repeat and fibronectin type III domain containing 2
	*PAPPA2*	4.466694057	4.32E-08	pappalysin 2
	*LINC00355*	4.362773601	2.47E-07	long intergenic non-protein coding RNA 355
	*CD79B*	4.319425344	4.58E-09	CD79b molecule
	*ADAMTS14*	4.137978722	1.17E-04	ADAM metallopeptidase with thrombospondin type 1 motif 14
	*GIPR*	3.873824073	8.49E-06	gastric inhibitory polypeptide receptor
	*GAST*	3.843110236	1.41E-06	gastrin
	*TMEM132B*	3.808227234	3.24E-06	transmembrane protein 132B
Down	*UGT2A1*	-7.360646644	1.08E-22	UDP glucuronosyltransferase family 2 member A1 complex locus
	*LIPF*	-7.162552451	4.49E-20	lipase F, gastric type
	*KRT40*	-6.971593921	5.43E-18	keratin 40
	*LHB*	-6.904734759	8.62E-24	luteinizing hormone subunit beta
	*CRYM*	-6.59095811	1.72E-14	crystallin mu
	*FSHB*	-6.551217182	7.35E-16	follicle-stimulating hormone subunit beta
	*KLK12*	-5.923450985	1.72E-14	kallikrein related peptidase 12
	*GPC5*	-5.749748325	1.99E-24	glypican 5
	*GPC4*	-5.652677963	1.41E-13	glypican 4
	*GAL*	-5.573472519	1.78E-12	galanin and GMAP prepropeptide

Log2FC represents log 2-fold change.

### 3.3 Differentially enriched pathways in GH-secreting pituitary tumors compared to normal pituitaries

To delve deeper into the functions of DEGs, we utilized GO analysis to annotate these DEGs and examine their distribution. Compared to normal pituitary glands, pathways involved in mitosis and cytokinesis were elevated in GH-secreting pituitary tumors (**Figure 1C**). Pathways integral to developmental processes (such as endocrine system development, pituitary gland development, and neuroendocrine cell differentiation) and hormone secretion for the pituitary gland were significantly suppressed in GH-secreting pituitary tumors. Additionally, the pathways governing ERK1/2, Wnt, and Notch signaling were consistently downregulated in somatotroph adenomas.

KEGG pathway enrichment analysis validated a significant enhancement in the pathways for cell cycle and motor protein in GH-secreting pituitary tumors (**Figure 1D**). Conversely, the neuroactive ligand-receptor interaction pathway appears to be suppressed in somatotroph adenomas. Furthermore, the signaling pathways involving PI3K-Akt, calcium, cAMP, Wnt, and TGF-beta exhibit significant downregulation in somatotroph adenomas compared with that in normal pituitaries.

### 3.4 Transcriptional signature of SGST compared to DGST

**Figure 2:**
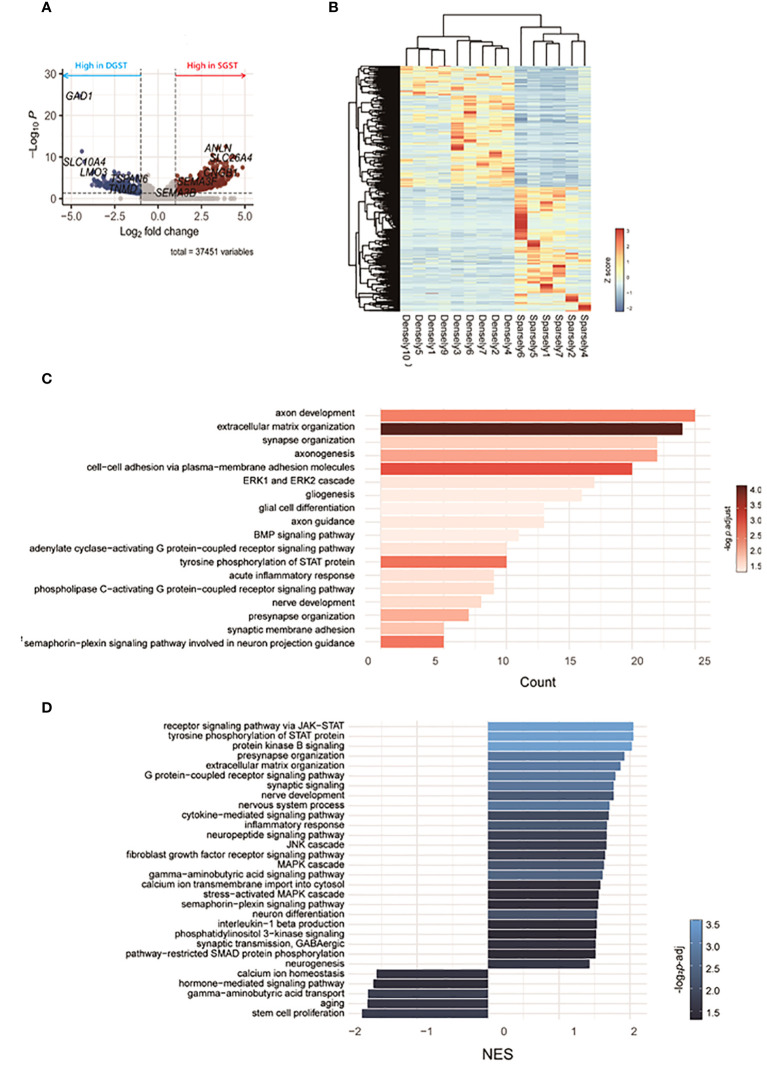
Transcriptomic differences between SGST and DGST. **(A)** Volcano plot indicating the DEGs in SGST compared to those in DGST. **(B)** The heatmap indicates gene expression comparisons between SGST and DGST. The colors indicate log2FC. **(C)** The bar plot visualizes the results of the GO analysis. The x-axis indicates counts, and the bar color indicates the p-adjust value. **(D)** Bar plot visualizes the results of the enriched pathway by GSEA analysis. The x-axis indicates normalized enrichment scores (NES), and the bar color indicates the p-adjust value.

RNA sequencing revealed differences in gene expression between GH-secreting pituitary tumors based on granulation patterns (**Figure 2**). A list of representative DEGs in SGST compared to DGST is provided in **Table 3**. **Figure 2A** illustrates a volcano plot of the genes differentially regulated in SGST vs DGST. This analysis identified 680 upregulated and 437 downregulated genes in SGST compared with that in DGST.

**Table 3:** Representative DEGs in SGST compared to DGST.

Condition	Gene	Log2FC	Adjusted P value	Description
Up	*ADAMTS14*	5.120722831	1.07E-08	ADAM metallopeptidase with thrombospondin type 1 motif 14
	*MPPED1*	4.86941283	3.27E-08	metallophosphoesterase domain containing 1
	*KCNQ5*	4.411001745	1.08E-10	potassium voltage-gated channel subfamily Q member 5
	*SLC24A2*	4.399146006	2.57E-07	solute carrier family 24 member 2
	*EBF3*	4.354230111	7.77E-11	EBF transcription factor 3
	*NTNG1*	4.271713112	2.75E-08	netrin G1
	*LINC01993*	4.248622784	1.88E-06	long intergenic non-protein coding RNA 1993
	*KCNV1*	4.19328269	3.78E-07	potassium voltage-gated channel modifier subfamily V member 1
	*GRIK3*	4.190680437	4.42E-07	glutamate ionotropic receptor kainate 3
	*SLC26A4*	4.189338263	1.33E-10	solute carrier family 26 member 4
Down	*GAD1*	-4.555869312	1.90E-25	glutamate decarboxylase 1
	*SLC10A4*	-4.264144115	1.37E-09	solute carrier family 10 member 4
	*TMEM45B*	-3.826010499	9.63E-07	transmembrane protein 45B
	*CALCA*	-3.81316813	5.41E-04	calcitonin-related polypeptide alpha
	*LMO3*	-3.728220598	3.86E-07	LIM domain only 3
	*PLP1*	-3.699205089	2.43E-05	proteolipid protein 1
	*TRPC7*	-3.672755858	6.89E-05	transient receptor potential cation channel subfamily C member 7
	*CALCB*	-3.568832742	6.50E-04	calcitonin-related polypeptide beta
	*STAR*	-3.563921939	7.61E-05	steroidogenic acute regulatory protein
	*PAQR9-AS1*	-3.525323167	8.81E-05	PAQR9 antisense RNA 1

Log2FC represents log 2-fold change.

Gene sets significantly enriched in SGST, as determined using GO analysis, are presented in **Figure 2C**. Eighteen pathways were found to be significantly enriched in SGST, including those involved in neuronal development (axon development, axonogenesis, axon guidance, synapse and presynapse organization, gliogenesis, glial cell differentiation, nerve development, synaptic membrane adhesion, semaphorin-plexin signaling pathway involved in neuron projection guidance) and acute inflammatory response.

No pathways were significantly downregulated in SGST compared with those in DGST.

To further assess the differences attributable to granulation patterns, we conducted GSEA. In SGST, 25 gene sets were significantly enriched, whereas 5 gene sets were enriched in DGST (**Figure 2D**). Signal pathways including JAK-STAT, protein kinase B, GPCR, JNK, fibroblast growth factor receptor, MAPK, and phosphatidylinositol 3-kinase are upregulated in SGST. Similar to the result of GO analysis, SGST showed enhancement of gene sets for both neuronal development (presynapse organization, synaptic signaling, nerve development, nervous system process, neuron differentiation, and neurogenesis) and inflammation (cytokine mediated signaling pathway, inflammatory response, interleukin 1 beta production). DGST showed differentially enriched categories of calcium ion homeostasis, hormone mediated signaling pathways, GABA transport, aging and stem cell proliferation.

**Figure 3:**
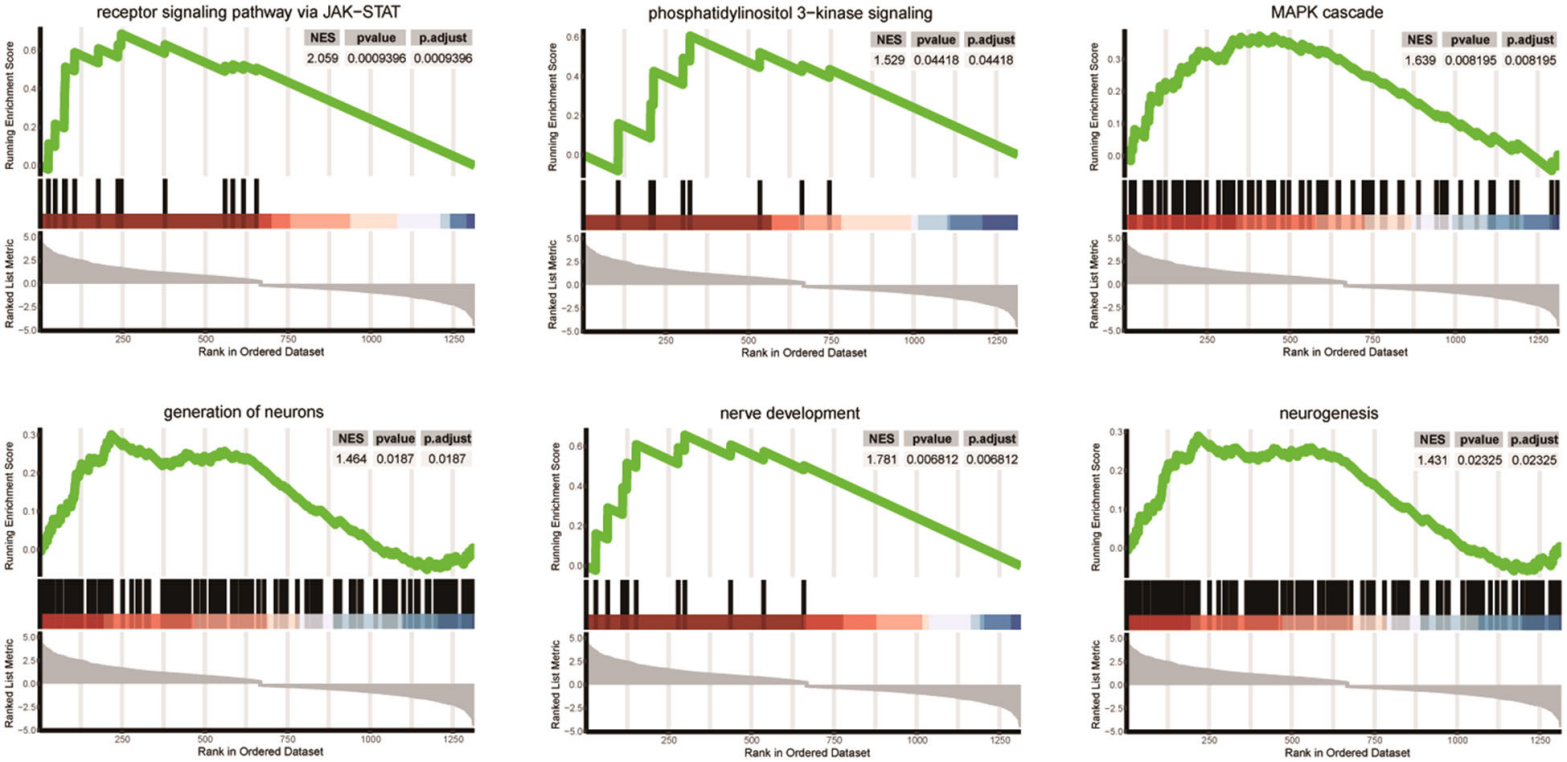
Differentially enriched pathways between SGST and DGST. Each plot shows NES, p-value, and p-adjust value. Enrichment plots for data sets commonly enriched in both GO and GSEA analysis showing the profile of the running enrichment score and positions of gene set members on the rank-ordered list.

We presented significantly enriched categories in both GO and GSEA analyses in **Figure 3**. Three signaling pathways (JAK-STAT, phosphatidylinositol 3-kinase, and MAP cascade) and three neuronal development processes (generation of neurons, nerve development, and neurogenesis) were significantly enriched.

### 3.5 PPI network of DEGs between SGST and DGST

**Figure 4:**
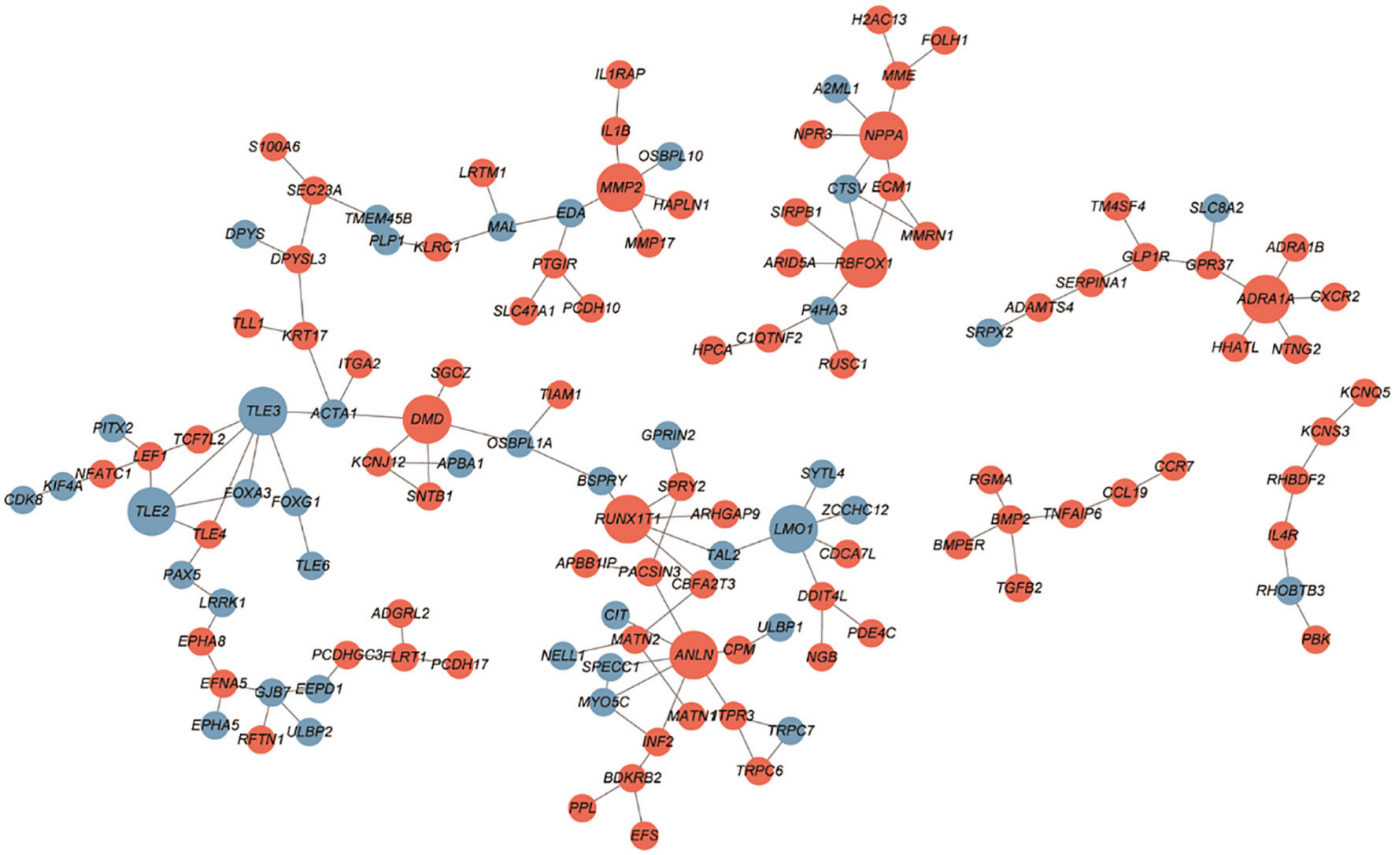
PPI network between SGST and DGST. PPIs were indicated by the network using cytoscape software. The nodes indicate DEGs in SGST compared to DGST, and the strings indicate the interaction between two proteins. The red and blue node indicate up and downregulated DEGs, respectively.

The PPI networks constructed with the up- and downregulated DEGs consisted of 128 and 132 PPI pairs, respectively (**Figure 4**). There were 10 hub proteins, top highly connected DEGs: *ANLN*, *TLE3*, *TLE2*, *DMD*, *ADRA1A*, *RBFOX1*, *MMP2*, *LMO1*, *RUNX1T1*, and *NPPA*.

## 4 Discussion

In this study, we present, for the first time, data on gene expression variations in GH-secreting pituitary tumors according to their granulation patterns and identify differential pathways that may underpin the distinct clinical characteristics of these patterns. In order to understand the diversity within the disease group of acromegaly, many transcriptomic approaches have been conducted (13). One recent study proposed classifying GH-secreting tumors into three groups through transcriptomic profiling, with the key classification being the presence of *GNAS* mutation and granulation pattern (32). While there has been extensive research on the characteristics of somatotroph tumors based on the presence of *GNAS* mutation, studies focusing on granulation are scarce (33, 34). Papers reporting on transcriptomic expression based on granulation pattern are mainly limited to methods utilizing targeted sequencing (14). Our results could be contribute to understand heterogeneity of acromegaly and lead to the development of potentially more effective therapeutic targets by suggesting important pathways and novel targets based on granulation patterns.

We observed that SGST showed an upregulation in inflammatory response in both GO and GSEA analysis compared to DGST. Tumor invasion of surrounding tissues, a hallmark of aggressive behavior, is intricately connected to the inflammatory response pathway (35, 36). Various components, including matrix metalloproteinases (MMP), urokinase plasminogen activator, membrane-type 1 MMP (MT1-MMP), cathepsin B, caspase-B, integrin, disintegrin, and metalloproteinases, are crucial for normal leukocyte migration. These components are also involved in managing the invasive traits of cells and tumor progression (37, 38). Previous studies have reported the role of MMPs in invasive and recurrent pituitary adenomas (39, 40). However, the significance of inflammatory response pathways in pituitary tumors is not yet fully understood. Similar to other types of cancer, these pathways may be instrumental in the aggressive behavior of somatotroph adenomas. Therefore, further research is warranted to explore this connection.

Our data also reveal that gene sets related neuronal development and neurotransmitters are dysregulated in SGST. Previous studies suggested neural environment surrounding tumors are important in cancer progression and neurotrophic factors from cancer cells is implicated in cancer progression by facilitating the infiltration of nerve cells into the tumor microenvironments (41). This holds true not only for neuronal tumors but also for prostate, colorectal or lung cancers (42–44). The expression of players in the neural circuits of somatotroph adenomas could serve as potential biomarkers for tumor progression.

Our data propose a range of novel transcriptomic elements that may be responsible for the characteristics of SGST: *EBF3*, *NTNTG1*, *GRIK3*, *KCNQ5*, *KCNV1*, *ADAMTS14*, *MPPED1*, and *SLC24A2*.

The early B-cell factors (EBFs) are transcription factors with *EBF3* playing dual oncogenic roles, depending on the cancer type, and linked to poor outcomes in various cancers (45, 46). This study suggests *EBF3*’s potential oncogenic effects in GH-secreting pituitary tumors. Nectrin G1 (*NTNG1*) aids in cell adhesion and axon guidance, and is implicated in cisplatin resistance and tumorigenesis (47, 48). Glutamate receptor kainate 3 (*GRIK3*) promotes epithelial-mesenchymal transition (EMT) in cancers, correlating with poor prognosis, and is overexpressed in GH-secreting pituitary tumors, indicating potential aggressiveness (49, 50). Additionally, voltage-gated ion channels and genes like *KCNQ5*, *KCNV1*, *ADAMTS14*, *MPPED1*, and *SLC24A2* are linked to tumor traits in these tumors (51).

We identified that the JAK–STAT signaling pathway is upregulated in SGST compared to DGST. This pathway is critical in cancer progression, acting as a primary driver of tumor growth and metastasis or as a modulator of immune surveillance (52). Within the STAT family, *STAT3* is particularly crucial in regulating gene expression associated with cancer progression. It can increase the expression of genes that promote anti-apoptosis, angiogenesis, metastasis, and cell cycle progression while also decreasing the expression of growth-suppressing genes. Immunohistochemistry has revealed that GH-producing pituitary adenomas express high levels of *STAT3* (53). As a transcriptional factor in these adenomas, *STAT3* regulates several genes that control hormone regulation and cell proliferation. *In vitro* studies have demonstrated that *Stat3* downregulation leads to heightened GH production in GH4 cells (54). Conversely, an increase in *Stat3* elevates GH expression and diminishes PRL expression in GH3 cells, with overexpression of *Stat3* boosting proliferation (53). The *STAT3* inhibitor S3I-201 decreases the growth of GH-producing pituitary adenoma cells both *in vitro* and *in vivo* in a concentration-dependent manner (53). However, more research, ranging from fundamental to clinical studies, is required to ascertain the importance of the JAK–STAT pathway in GH-secreting pituitary adenomas. Further investigations using animal models and cellular assays involving knockout or overexpression of specific transcripts may confirm the essential changes in the characteristics of SGST.

Through PPI network we revealed three downregulated hub DEGs (*TLE2*, *TLE3*, and *LMO1*) and seven upregulated hub DEGs (*DMD*, *MMP2*, *NPPA*, *ADRA1A*, *RBFOX1*, *RUNX1T1*, and *ANLN*). Especially *MMP2* is involved in tissue inflammation and neurodegeneration, while *ANLN* is involved in cell division and cell movement (55, 56). Both of these genes are involved in inflammatory responses and are upregulated in SGST, which is consistent with the result that the inflammatory response gene set is enhanced in SGST in the GO analysis.

There were some limitations to our study. First, CAM5.2 staining was not verified in this study. A previous study recommended CAM5.2 staining to evaluate subgroups of somatotroph adenomas (57–59). Although no significant differences in clinical characteristics were observed based on the granulation pattern in our sequencing cohort, re-evaluating the granulation pattern through CAM5.2 staining may prove helpful. In addition, we did not test *GNAS* mutations for the tumors included in this study. Previous studies have suggested that the *GNAS* mutation is an important factor in transcriptomic profiling, with reports indicating that approximately 35–59% of Korean patients are *GNAS*-positive (60, 61). However, current research mostly finds no difference in *GNAS* mutations based on granulation patterns (12, 62). Nevertheless, gene expression based on granulation patterns may be affected by the presence or absence of *GNAS* mutations, warranting further investigation in future research.

In this study, we delineated a transcriptional signature depending on the granulation pattern of somatotroph adenomas. These findings reveal a range of new transcriptional alterations that could be instrumental in the varied clinical characteristics observed in patients with acromegaly, depending on their granulation pattern.

## Data availability statement

The datasets presented in this study can be found in online repositories. The names of the repository/repositories and accession number(s) can be found in the article/supplementary material.

## Ethics statement

The studies involving humans were approved by Ethics Committee of Severance Hospital. The studies were conducted in accordance with the local legislation and institutional requirements. The participants provided their written informed consent to participate in this study. Ethical approval was not required for the studies on animals in accordance with the local legislation and institutional requirements because only commercially available established cell lines were used.

## Author contributions

KK: Conceptualization, Data curation, Formal analysis, Investigation, Methodology, Validation, Visualization, Writing – original draft, Writing – review & editing. YK: Data curation, Formal analysis, Methodology, Visualization, Writing – original draft. SK: Methodology, Resources, Writing – review & editing. JM: Resources, Writing – review & editing. EK: Resources, Writing – review & editing. EL: Resources, Writing – review & editing. C-MO: Methodology, Resources, Supervision, Writing – review & editing. CK: Conceptualization, Funding acquisition, Project administration, Supervision, Writing – original draft, Writing – review & editing.
